# A national pharmacovigilance centre perspective on pandemic preparedness - *lessons learned from the COVID-19 pandemic*

**DOI:** 10.3389/fdsfr.2025.1644680

**Published:** 2025-08-15

**Authors:** Florence van Hunsel, Agnes Kant

**Affiliations:** ^1^ Netherlands Pharmacovigilance Centre Lareb, ’sHertogenbosch, Netherlands; ^2^ Department of PharmacoTherapy—Epidemiology and Economics, Groningen Research Institute of Pharmacy (GRIP), University of Groningen, Groningen, Netherlands; ^3^ Department of Clinical Pharmacology and Toxicology, Leiden University Medical Centre, Leiden, Netherlands

**Keywords:** pandemic preparedness, pharmacovigilance, COVID-19 vaccines, adverse drug reaction (ADR), adverse event following immunization (AEFI)

## Abstract

The SARS-CoV-2 (COVID-19) pandemic highlighted the critical role of pharmacovigilance in ensuring vaccine and drug safety. This perspective from the Netherlands Pharmacovigilance Centre Lareb outlines key experiences and lessons learned during the pandemic. Lareb managed over 233,000 individual case safety reports (ICSRs) related to COVID-19 vaccines, with a considerable proportion submitted by consumers/vaccinated persons directly. Lareb employed both spontaneous reporting and cohort event monitoring (CEM) to gain a better understanding of the safety of these vaccines in a real-world setting. Challenges included the overwhelming volume of data, limited initial access to national vaccination and healthcare registries, underreporting of adverse reactions to SARS-CoV-2 treatments, and a strain on the trained staff to perform tasks while scaling up in personnel. Lareb addressed some challenges through further automation, although more work in this area is still needed. Communication efforts were expanded with a focus on transparency and timeliness. Key recommendations for future pandemic preparedness include investing in Artificial Intelligence for further automation in the reporting process and in signal detection, looking at ways to tackle underreporting for specific associations or medicines in innovative ways and enhancing timely linkage between vaccination and healthcare data. The article underscores the importance of transparent, independent communication and the need for a resilient pharmacovigilance system capable of rapid scale-up during health crises.

## 1 Introduction

The COVID-19 pandemic has underscored the critical importance of robust pharmacovigilance systems. Pharmacovigilance, defined as *‘the science of detecting, assessing, understanding, and preventing adverse effects or any other drug-related problems’* (European Medicines Agency, 2022), played a pivotal role during the COVID-19 pandemic (Rudolph et al., 2022). The rapid development and deployment of COVID-19 vaccines necessitated an unprecedented level of safety monitoring.

In the Netherlands, key national stakeholders such as the Medicines Evaluation Board (MEB), the National Institute for Public Health and the Environment (RIVM) and the Netherlands Pharmacovigilance Centre Lareb collaborated to monitor the safety of vaccines and treatments. The MEB is the national competent authority in the Netherlands, responsible for medicine marketing authorisations. Lareb is the reporting and knowledge centre for adverse drugs reactions (ADRs) and maintains the spontaneous reporting system (SRS) in the Netherlands. Analysis of ADR reports submitted to Lareb can lead to identification of risks associated with the use of medicines in daily practice. Signals about previously unknown (aspects of) ADRs are then disseminated to MEB who can take autonomous regulatory actions or forward the signal for further evaluation to the Pharmacovigilance Risk Assessment Committee (PRAC) at the European Medicines Agency (EMA) or lead member states responsible for nationally authorised products (van Hunsel et al., 2021). In addition to the SRS, Lareb maintains a system for cohort event monitoring (CEM) studies, which is employed in many studies on vaccine- and drug safety. Lareb is also specialized in drug use during pregnancy and lactation with the Dutch Teratology Information Service (TIS). The Dutch Pregnancy Drug Register monitors the use and safety of drugs during pregnancy and lactation and Lareb performs research on this data. In this article, we reflect on the lessons learned at Pharmacovigilance Centre Lareb during the COVID-19 pandemic. Based on a previous in-depth evaluation performed by Lareb (RSNN, 2024) we will give insight in the challenges faced by a national pharmacovigilance centre and describe actionable recommendations to improve the pharmacovigilance system in the Netherlands.

## 2 Highlights of Lareb’s role during the COVID-19 pandemic

### 2.1 Spontaneous reporting system

**FIGURE 1:**
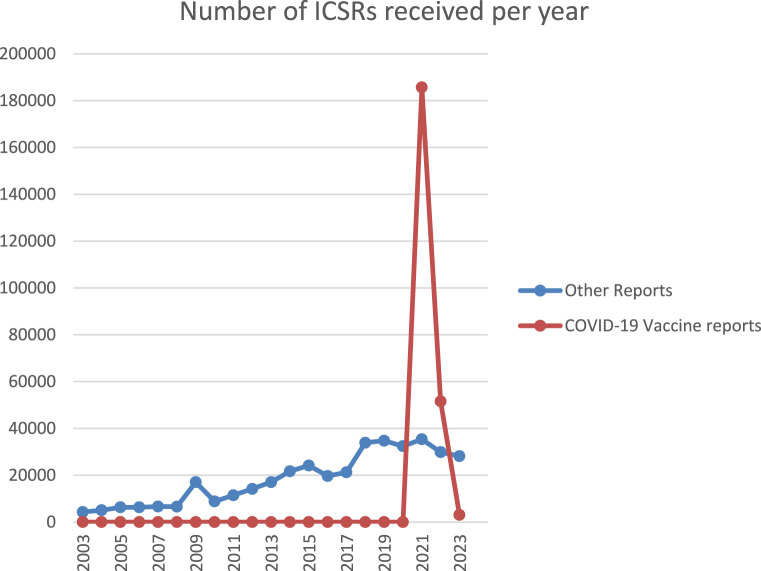
Overview of the ICSRs received in the period 2003‐2023, with the reports on COVID‐19 vaccines shown separately from reports on other medicines.

**FIGURE 2:**
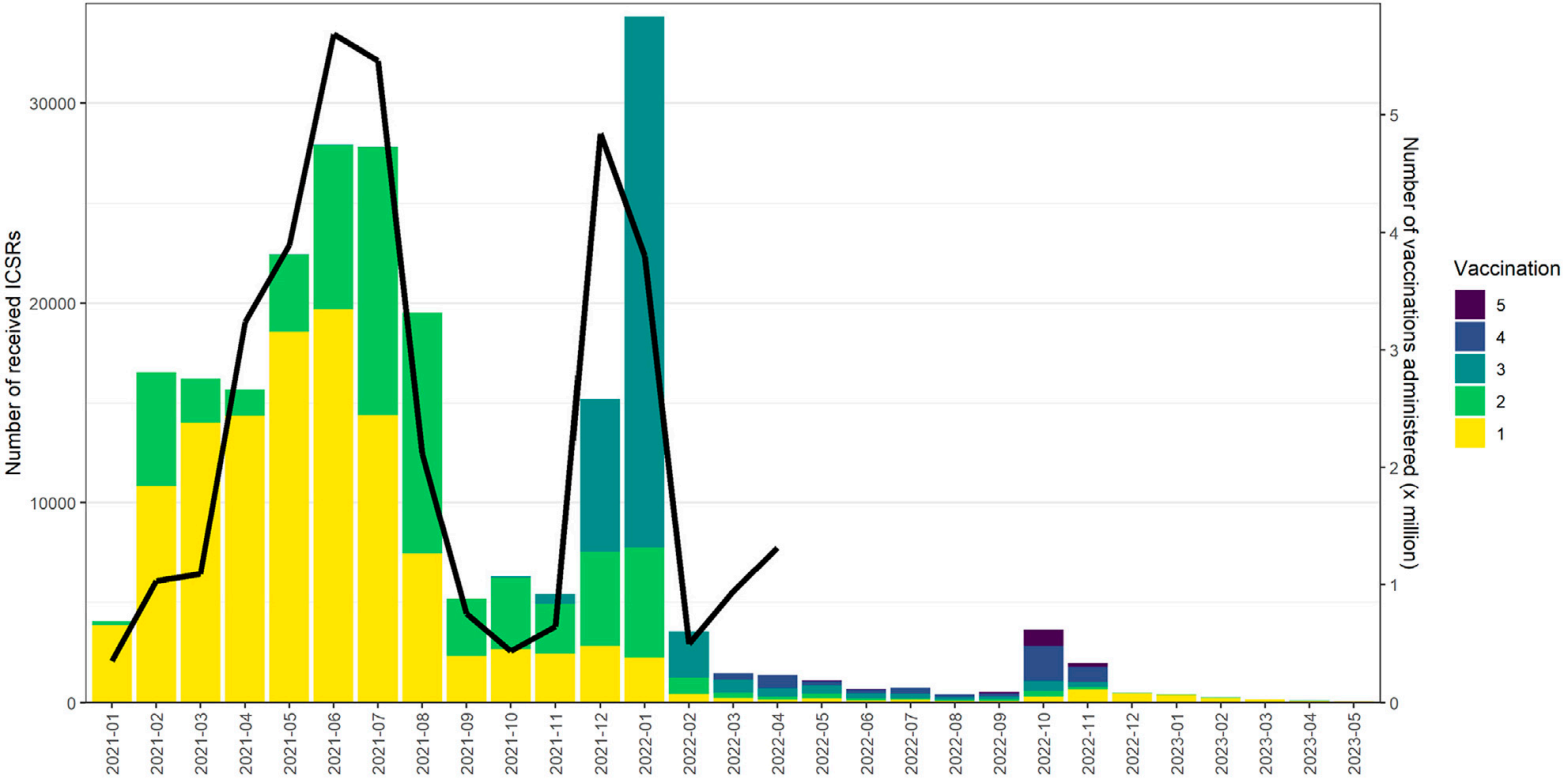
The number of ICSRs received per dose vs. the number of vaccinations administered in the Netherlands.

Lareb collects around 30,000 Individual Case Safety Reports (ICSRs) of ADRs yearly. The pandemic saw a significant increase in the number of ICSRs to the Dutch SRS. See Figure 1 for an overview of the ICSRs received in the period 2003–2023, with the reports on COVID-19 vaccines shown separately from reports on other medicines. The number of ICSRs received, stratified by vaccination dose, was analysed until May 2023 for the previously mentioned evaluation of Lareb’s work during the pandemic (RSNN, 2024). At that time, Lareb had received over 233,428 ICSRs related to COVID-19 vaccines with over a million ADRs reported. Most reports came from consumers and non-healthcare professionals, with 114,968 ICSRs being reported after the first dose, 66,402 after the second dose, and 39,574 after the third dose. For the remainder of the reports the dose number was unknown. The percentage of reports with serious outcomes (defined by international criteria) ranged from 1.6% to 2.9% across different vaccination doses. Figure 2 shows the number of ICSRs received per dose vs. the number of vaccinations administered in the Netherlands. It should be noted that data on the number of vaccinations administered was available to Lareb until April 2024. Figure 2 shows that peaks in the number of ICSRs follow mass vaccination moments in the Netherlands.

This surge in data required efficient processing and analysis to identify potential safety signals (Oosterhuis et al., 2023). Analysis methods also included making more use of background rates (Sturkenboom et al., 2022) in Observed vs. Expected analysis for various outcomes such as thrombosis, myo- and pericarditis and Bell’s palsy (van der Boom et al., 2023). Volumes of ICSRs received on some vaccine-event associations were exceptionally high; For instance, from 6 January 2021, to 1 December 2021, Lareb received 17,735 ICSRs of menstrual disorders and postmenopausal bleeding after vaccinations with AstraZeneca, Johnson and Johnson, Moderna, and Pfizer vaccines (Duijster et al., 2023a).

In a biweekly meeting key features of incoming reports and potential signals were discussed with the MEB and the RIVM. If needed there were additional *ad hoc* discussions on signals planned. The pharmacovigilance system successfully generated new knowledge (Kant et al., 2022a). For instance, analysis of the previously mentioned reports on menstrual disorders after COVID-19 vaccination led to safety signals to the MEB (Duijster et al., 2023a). In addition, signals were issued to the MEB on potential serious adverse reactions such as thrombosis and thrombocytopenia syndrome (TTS), thrombosis and Guillain-Barré syndrome, based on the Dutch number of cases and number of administrated vaccines in the Netherlands. A full overview of signals and other knowledge disseminated by Lareb during the pandemic is given in Tables 1, 2. This includes signals on COVID-19 vaccines and for medicines used in the treatment of outcomes related to SARS-CoV-2 infection. After evaluation by the MEB, signals could be discussed at PRAC, which is the EMA committee responsible for assessing and monitoring the safety of human medicines (Rudolph et al., 2022).

**TABLE 1:** Signals and other knowledge dissemination for COVID-19 vaccines 2020–2024.

1. Thrombosis after COVID-19 vaccination
2. Myocarditis and pericarditis after COVID-19 vaccination
3. Autoimmune haemolytic anaemia after COVID-19 vaccination
4. Enlarged lymph nodes after COVID-19 vaccination
5. Guillain-Barré syndrome after COVID-19 vaccination
6. COVID-19 vaccine during the breastfeeding period
7. Loss of smell and taste after COVID-19 vaccination
8. Menstrual disorders after COVID-19 vaccination
9. Long COVID symptoms after COVID-19 vaccination
10. Overview of transverse myelitis after COVID-19 vaccination
11. ADRs after COVID-19 vaccination in people with multiple sclerosis (MS)
12. Guillain-Barré Syndrome after COVID-19 vaccination
13. Women more often experience ADRs after COVID-19 vaccination than men
14. Pregnant women have known and expected ADRs after COVID-19 vaccination
15. Reports of thyroid dysfunction after COVID-19 vaccination
16. More insight needed about ‘Long COVID’ complaints after COVID-19 vaccination
17. Neuralgic amyotrophy after COVID-19 vaccination
18. Heavy menstrual bleeding in SmPC Pfizer and Moderna COVID-19 vaccine
19. Allergic reactions mainly reported after the first COVID-19 vaccination
20. Skin reactions in tattoos after COVID-19 vaccination
21. Headaches are common after COVID-19 vaccination
22. No indications of adverse outcomes of COVID-19 vaccination in pregnant women
23. Myocarditis an pericarditis ADR of Novavax COVID-19 vaccine
24. Reports of Bell’s facial palsy after COVID-19 vaccination
25. Skin condition lichen planus reported after COVID-19 vaccination
26. Nerve disorder neuralgic amyotrophy after vaccination
27. More research into menstrual disorders after COVID-19 vaccination
28. Overview of reports of deaths after COVID-19 vaccination
29. Update of reports of myocarditis and pericarditis after COVID-19 vaccinations
30. Update of reports of thromboses and embolisms after COVID-19 vaccinations
31. Update of reports of thrombosis with a low platelet count after COVID-19 vaccination
32. Deceased breast milk is reported after COVID-19 vaccination
33. Many people experience ADRs after COVID-19 vaccination
34. Persistent shoulder complaints after vaccination
35. Cutaneous vasculitis after Janssen COVID-19 vaccination
36. Flare capillary leak syndrome after Moderna COVID-19 vaccination
37. Glucose fluctuations in diabetic patients after COVID-19 vaccination
38. Enlarged lymph nodes occur relatively more often after COVID-19 booster vaccinations
39. Spinal cord inflammation (Myelitis Transversa) rare ADR of AstraZeneca and Janssen COVID-19 vaccine
40. Scar reactivation of old BCG-vaccination scars after Moderna COVID-19 vaccine
41. Hypersensitivity reactions on dermal filler after AstraZeneca COVID-19 vaccine
42. Menstrual disorder possibly a possible ADR after COVID-19 vaccinations
43. Less reported cases of severe allergic reactions after a second COVID-19 vaccination
44. Overview of reports on myocarditis and pericarditis after COVID-19 vaccination
45. Cerebral venous sinus thrombosis after AstraZeneca COVID-19 vaccine
46. Vasculitis reported after COVID-19 vaccination
47. Immune thrombocytopenia (ITP) after AstraZeneca and Janssen vaccination
48. Venous thrombosis included in SmPC after Janssen vaccination
49. Hugh amounts of received reports of menstrual disorders following COVID-19 vaccination
50. Research on venous thrombosis after Janssen COVID-19 vaccination
51. Reports of Bullous Pemphigoid after Pfizer/BioNTech COVID-19 vaccination
52. Guillain–Barré syndrome after vaccination with AstraZeneca COVID-19 vaccine
53. Cases of thrombosis with low platelet count after COVID-19 vaccination
54. Myelitis Transversa after vaccination with AstraZeneca COVID-19 vaccine
55. Myocarditis and pericarditis after vaccination with Pfizer- and Moderna COVID-19 vaccine
56. Contraindication with Janssen COVID-19 vaccine for patients with previous systemic capillary leak syndrome
57. Overview of reports with fatal outcome after COVID-19 vaccination
58. Research on myocarditis after COVID-19 vaccination
59. Contraindication with the AstraZeneca COVID-19 vaccine for patients with previous systemic capillary leak syndrome
60. Contraindication with the AstraZeneca COVID-19 vaccine for patient with previous thrombosis in combination with low platelet count
61. More research needed to investigate thrombosis and thromboembolism after COVID-19 vaccination
62. Thrombosis with low platelet count eight times reported after COVID-19 vaccination
63. First overview of reports with fatal outcome after COVID-19 vaccination
64. Reports of thrombosis and low platelet count after vaccination with the AstraZeneca COVID-19 vaccine
65. Extensive limb swelling (ELS) after the Pfizer/BioNTech vaccine
66. COVID-19 vaccination and thrombosis

**TABLE 2:** Signals and other knowledge dissemination for medicines for SARS-CoV-2 infection or SARS-CoV-2 infection during pregnancy 2020–2024.

1. Respiratory problems with treatment casirivimab/imdevimab for SARS-CoV-2 symptoms
2. Hepatic and renal impairment with remdesivir
3. Reports adverse drug reactions of hydroxychloroquine and chloroquine
4. Serious adverse drug reactions of (hydroxy) chloroquine in SARS-CoV-2 patients
5. Adverse drug reactions with drug treatment for SARS-CoV-2 infection (COVID-19)
6. SARS-CoV-2 infection (COVID-19) during pregnancy
7. Paracetamol first choice to suppress fever in patients with SARS-CoV-2 symptoms

### 2.2 Cohort event monitoring

In addition to the SRS, Lareb conducted a large CEM study. This study using patient-reported outcomes (PROs) in the Netherlands started in February 2021 and used a prospective cohort design. CEM is considered active surveillance and allows for in-depth information on the course of reported ADRs, with a denominator allowing for risk quantification. It is well suited to capture reactogenic events, including those that are not medically attended, and which vaccinated persons can report themselves. The value of CEM as complement to the existing SRS is that it can give insight into the incidence of adverse reactions in a large group of vaccinated persons, followed over a period of months, and give information on the time-to-onset, duration, treatment, risk groups and burden of reported events. These aspects can be under-represented in pre-authorization studies (Kant et al., 2022b; Rolfes et al., 2022; Duijster et al., 2023b). The Dutch CEM study included over 27,000 vaccinees. Common adverse events following immunization (AEFI) - such as headache, fatigue, muscle and joint pain, fever, and chills—were frequently reported after COVID-19 vaccination. Corrected for the proportion of the different vaccine brands used in the cohort, AEFI were most prevalent after the first dose of AstraZeneca (Vaxzevria) and Janssen (Jcovden), and after both doses of Moderna (Spikevax). Pfizer/BioNTech (Comirnaty) was associated with fewer AEFI overall. Women and younger individuals reported AEFI more often. Prior SARS-CoV-2 infection increased the likelihood of AEFI after the first dose of any vaccine, and after the second Moderna dose. Most AEFI appeared within 14 h of vaccination and resolved within a few days, regardless of vaccine type or dose. Based on a common protocol (EU PAS Register Number EUPAS39798), data could be combined with similarly collected data in other European countries. Both aggregated data (Raethke et al., 2023) and combined data, through a common data model (Raethke et al., 2024), were analysed. In this way it was possible to monitor the safety of first, second, and booster doses of EMA-approved COVID-19 vaccines in the general population and special populations such as patients with allergy (Luxi et al., 2024) and immune-compromised patients (Bellitto et al., 2024). Booster vaccinations, primarily with Pfizer and to a lesser extent Moderna, followed similar patterns, with women and younger recipients more frequently affected (Raethke et al., 2024).

### 2.3 Vaccination during pregnancy

Information regarding the risk of COVID-19 vaccination during pregnancy on the development of congenital anomalies was essential. Data from the Dutch Pregnancy Drug Register, an ongoing cohort study maintained by Lareb, could be used to study potential risks of vaccination during pregnancy and lactation. Based on these data no association was found between COVID-19 vaccination during pregnancy and the risk of miscarriage, preterm birth or congenital anomalies when vaccination occurred in the first trimester (Woestenberg et al., 2023; de Feijter et al., 2024; Woestenberg et al., 2025).

### 2.4 Knowledge centre

Knowledge on drug and vaccine safety is crucial for healthcare professionals and patients for the prevention, recognition, and treatment of ADRs and to make informed choices. Moreover, robust monitoring of vaccine safety and transparency of (potential) ADRs is essential for maintaining public trust, especially in times when there are many questions and concerns about vaccination. To support this, Lareb provides up-to-date easily accessible information on its website (www.lareb.nl). As shown in Tables 1, 2 Lareb disseminated a total of 66 signals and other new knowledge about COVID-19 vaccines and seven about medicines for the treatment of SARS-CoV-2 infection in the period 2020–2024. Information about safety of the COVID-19 vaccines was shared online. First weekly updates appeared and later biweekly updates. Lareb also maintains an online knowledge bank containing information on both known and alleged ADRs of vaccines that was expanded during the pandemic (Kant et al., 2022a). In addition, Lareb also provided information through various social media platforms, via a dedicated telephone service for healthcare professionals and the public and through media appearances.

## 3 Challenges during the COVID-19 pandemic

### 3.1 Large volume of data vs. underreporting

Lareb already automated many steps in the reporting process before the start of the vaccination campaign. For instance, a specific COVID-19 vaccine-dedicated web-based reporting form was developed that enabled the collection of spontaneously reported information on the vaccine administered, suspected ADRs and other information needed for assessment and signal detection. As much information as possible was automatically coded and processed (Oosterhuis et al., 2023). However, the sheer volume of data necessitated even more efficient systems to handle the influx of information. In both the ICSR reporting form and the cohort event monitoring study, Lareb used a list of pre-specified AEFI which was based on the most commonly listed reactions in the Summary of Product Characteristics (SmPCs) of the COVID-19 vaccines. Pre-specified AEFI were for instance various injection site reactions, pyrexia, myalgia, arthralgia and headache. The pre-defined AEFI in the SRS and CEM study could be automatically coded to the Medical Dictionary of Regulatory Activities (MedDRA^®^). Other information such as dose number or information on a previous SARS-CoV-2 infection was automatically mapped to corresponding ICH e2B(R3) fields in the ICSR management system. Next to the pre-specified reactions, the reporter could choose an option to provide other AEFIs as free text. These AEFI had to be coded manually by trained staff (Oosterhuis et al., 2023). Because the coding was performed by a large group of coders, additional checks and efforts had to be made to maintain consistency in appointed codes. Unfortunately, Lareb’s previously developed auto-coding algorithm did not have the correct performance for coding all other reported adverse reactions.

In 2021, the year that the majority of COVID-19 vaccine ICSRs came in, over 33% of ICSRs could be handled in a fully automated manner. The rest of the incoming ICSRs had to be triaged on a daily basis by highly trained vaccine assessors to identify the reports that needed a priority clinical review (Oosterhuis et al., 2023). Triage was aimed at recognizing the ADRs with the most severe outcomes and potential signal value first. For certain focus areas, such severe allergic reactions, triage was also aimed at assigning ICSRs to assessors specializing in checking whether the cases adhered to the case definitions of the Brighton Collaboration and asking follow-up if information in the cases was deemed incomplete (Gold et al., 2023; Gold et al., 2010).

Once a potential signal was being analysed further, it was also challenging to select those cases with the highest level of (clinical) information due to volume of ICSRs. For instance, among over 17,000 ICSRs of menstrual disorders Lareb assessors had to manually screen for those cases where data was available on medical tests, the use of oral contraceptives, a medical history that could be related to menstrual disturbances, etc. For ICSRs where information was lacking, follow-up questions had to be sent to reporters and follow-up information added to the cases in a manual fashion. All these steps in the processing and analysis of ICSRs required a large increase in the number of pharmacovigilance staff.

Even though the volumes of ICSRs were very high, there could be a lack of important data in some areas. The majority of reports on COVID-19 vaccines came from vaccinated persons directly, who contributed to many of the signals issued by Lareb. However, also receiving ICSRs from healthcare professionals was deemed essential for many signals and underreporting is a known problem in SRS (Hazell and Shakir, 2006). In contrast to the high number of reports on COVID-19 vaccines, the number of reports on drugs to treat SARS-CoV-2 symptoms was very low with only 265 ICSRs being reported. The high workload of healthcare workers in the middle of the COVID-19 pandemic has likely been an important barrier in reporting ADRs.

### 3.2 Data on vaccination administration and data-linkage

At the beginning of the pandemic, no arrangements were present to establish the linkage with the national vaccination registry in the Netherlands (CIMS, maintained by the RIVM). Eventually, Lareb was granted access to this data through the RIVM. With the reporter’s consent, batch numbers and vaccine brands could be retrieved from the CIMS registry. This data was used to get the information in the SRS database as complete as possible. In addition, data from CIMS on the number of vaccinations administered in the Netherlands were provided by RIVM for signal detection activities, such as Observed vs. Expected analyses, where information on the number of vaccines administered—stratified by age and sex—is essential. Observed vs. Expected analyses became a standard analysis approach for COVID-19 vaccine signal detection, next to a clinical review of ICSRs, for events with a relatively high background rate.

It was not possible to link vaccination data with healthcare data in a fast and efficient manner in the Netherlands. This limitation hindered the timely evaluation of potential safety signals if needed. In contrast, many other EU member states were able to perform such linkages more effectively (Pottegård et al., 2021; Zureik et al., 2023; Ljung et al., 2021).

### 3.3 Communication

Pharmacovigilance plays a significant role in building society’s ‘substantiated trust’ in the safety of medicines and vaccines–this trust came under pressure during the pandemic. The existence of an independent reporting and knowledge center for ADRs proved to be extra important during the pandemic for the substantiated trust in medical knowledge on safety. In 2021 in total 12, 571 information requests on COVID-19 vaccines were answered through a dedicated telephone line for healthcare professionals and the public. Lareb actively contributed to 256 media items on COVID-19 vaccines, this includes interviews for radio, newspapers, and television. The website www.lareb.nl was updated with a dedicated section on COVID-19 vaccines. Lareb listed frequently asked questions on vaccines AEFI, showed up to date numbers of incoming ICSRs stratified for brands and made news items on all published signals and other generated knowledge. The number of website visitors rose from 742,215 in 2020 to 4,618,921 in 2021 (522% increase). These users visited over eleven million pages on the website during their visits, an increase of 380%. The new vaccine knowledge bank was consulted almost 1 million times. Also, the number of people who followed Lareb on various social media channels steeply increased. In 2021, 128 messages (+71% increase from 2020) were shared via Facebook, 145 (71% increase from 2020) via LinkedIn and 118 (+84% increase from 2020) via X (formally known as Twitter) (Lareb, 2021). The sheer volume of questions and the need for information from the public, healthcare professionals and journalists put a strain on the organisation, even though additional colleagues with a communication background were recruited.

## 4 Addressing challenges

### 4.1 Tackling large volume of data vs. underreporting

To be able to handle large volumes of data in the Dutch SRS more efficiently, we aim to utilize advanced automation techniques for processing and analyzing reports. Artificial intelligence (AI) is expected to play a crucial role in this regard (Dong et al., 2024). General‐purpose large language models (LLM) nowadays have the potential support a variety of applications such as auto-coding and text-mining (Correia Pinheiro et al., 2025). This can streamline the data processing workflow and improve efficiency. Methods have been developed to identify ADRs from unstructured data and code them (Létinier et al., 2021; Martin et al., 2022; Combi et al., 2018; Meldau et al., 2022) and to assist in the triage of cases (Gosselt et al., 2022; Lieber et al., 2023; Kara et al., 2023; Bergman et al., 2023). Lareb is currently working towards the employment of new methods in these areas. Learning from the experiences of other countries can provide valuable insights and best practices. Federated learning approaches (TNO, 2021), with the use of privacy-preserving data analysis techniques, could facilitate collaboration between centres in the development of new analysis tools suitable for large volumes of data.

To address the challenge of underreporting and quality of reports in the SRS, Lareb has undertaken several initiatives. In 2023, Lareb received funding through Netherlands Organisation for Health Research and Development (ZonMW) for a project focused on using electronic healthcare records (EHR) to fill knowledge gaps in vaccine safety surveillance during pandemics. This project was a collaboration between Lareb, the Leiden University Medical Center, and the Haga Hospital in The Hague. For potential new signals, targeted searches were performed in structured and unstructured EHR-data using a clinical data collector tool. The search criteria were based on information from spontaneous reports and scientific literature. Identified EHR-cases that after manual review turned out to endorse potential signals, were reported to the SRS of Lareb, which then analyzed these reports alongside spontaneously reported cases. This innovative approach to safety monitoring could be highly effective in future pandemics and has the potential to accelerate signal detection. The method is further described in a pilot study article (Abedian Kalkhoran et al., 2024).

During Lareb’s evaluation of work performed during the pandemic (RSNN, 2024), healthcare professionals have also expressed their wish that reporting from electronic healthcare systems directly to pharmacovigilance systems should be possible in a (semi-) automated manner to reduce the administrative burden on healthcare professionals and improve the quality of data collected. Reporting directly from electronic healthcare systems is possible in some countries such as the UK (England, 2024). Lareb has made extensive efforts for reporting from electronic health systems, but this is hampered by the large variety of different systems used in the Netherlands and lack of influence on development.

### 4.2 Data infrastructure

Looking ahead to future pandemics, it is important to look at the infrastructure that enables *timely* data linkages in the Netherlands. As the national pharmacovigilance centre, Lareb should have access and permission for linkage to vaccination registers without delay. Next to that, access to healthcare data and linkage with vaccination data should be possible, while fully adhering to EU privacy regulations. From 1 January 2022, to 31 December 2023, Lareb ran a pilot project, funded through the Ministry of Health, Welfare and Sport, in which an infrastructure was built to perform in depth analyses based on EHR data, among which the analysis on the risk of menstrual disorders (Jajou et al., 2024) and post-menopausal bleeding (Jajou et al., 2025) after COVID-19 vaccination. Retrospective self-controlled cohort studies were performed, based on patients registered in the General practitioner databases of Nivel (the Nivel Primary Care Database, Nivel-PCD) or PHARMO. The RIVM provided the vaccination data. The speed at which the studies could be performed was hampered by the previously mentioned data-linkage issues. For improving the timeliness of future studies in the Netherlands Health-RI (https://www.health-ri.nl), an organization working toward an integrated research infrastructure that facilitates the reuse of health data for policy, research, and innovation, could play a role. Within Europe, the Data Analysis and Real World Interrogation Network (DARWIN EU^®^) is also getting up to speed (Dernie et al., 2024). Hopefully, the European Health Data Space (EHDS) (European Commission, 2025) will also improve timely linkages between healthcare and vaccination data in all EU countries. The EHDS is a health specific ecosystem comprised of rules, common standards and practices, infrastructures and a governance framework aiming at empowering individuals through increased digital access to and control of their electronic personal health data, at national level and EU-wide. Secondly, it aims at fostering a single market for electronic health record systems, relevant medical devices and high risk AI systems and lastly, at providing a trustworthy and efficient set-up for the use of health data for research, innovation, policy-making and regulatory activities (secondary use of data) (European Commission, 2025).

## 5 Discussion and conclusion

The COVID-19 pandemic has underscored the necessity of robust pharmacovigilance systems to ensure the safety of vaccines and medicines (Matthew, 2024). Despite the challenges during the pandemic, the pharmacovigilance process was nevertheless carried out carefully, and the way pharmacovigilance is organised in the Netherlands, with different organisations with different roles, has proven to be functional. Even though the World Health Organisation (WHO) declared the end of COVID-19 as a public health emergency on May 5th of 2023 (Sarker et al., 2023), being prepared for a next pandemic is crucial. To be able to scale up in a brief time a sufficient base of qualified employees, who also can take responsibility for training new colleagues, is needed at Lareb. Clear, transparent, and independent communication on vaccine safety is vital to maintain public confidence. Providing timely and accurate information helps to build trust. However, this also means that adequate funding is needed to provide this information as it requires additional staff.

Gaps in the pharmacovigilance system included the limited visibility of the safety of medicines used (off-label) for the treatment of SARS-CoV-2 symptoms and the lack of a data infrastructure to quickly conduct follow-up research after finding suspected new ADRs through analysis of SRS and CEM data. By addressing the challenges as outlined above, the pharmacovigilance system in the Netherlands can enhance its preparedness for future pandemics.

## Data Availability

This article is based on a previous evaluation report on regulatory pandemic preparedness in pharmacovigilance in the Netherlands. This report is available through the Regulatory Science Network Netherlands: https://www.rsnn.nl/sites/rsnn/files/2023-12/Pandemic%20Preparedness%20-%20Pharmacovigilance%20FINAL%20Report.pdf.
